# Isolation of *Mycobacterium avium* ssp. *paratuberculosis* and other non-tuberculous mycobacteria from head lymph nodes of wild ruminants and badgers in Switzerland

**DOI:** 10.3389/fvets.2023.1321106

**Published:** 2024-01-04

**Authors:** Julia Lienhard, Ute Friedel, Claudio Paganini, Monika Hilbe, Simone Scherrer, Sarah Schmitt

**Affiliations:** ^1^Section of Veterinary Bacteriology, Vetsuisse Faculty, Institute for Food Safety and Hygiene, University of Zurich, Zurich, Switzerland; ^2^Office for Food Safety and Animal Health, Chur, Switzerland; ^3^Vetsuisse Faculty, Institute of Veterinary Pathology, University of Zurich, Zurich, Switzerland

**Keywords:** *Mycobacterium avium* ssp. *paratuberculosis*, non-tuberculous mycobacteria, red deer, wild ruminants, badger, retropharyngeal lymph node, mandibular lymph node, mycobacterial culture

## Abstract

**Introduction:**

The family *Mycobacteriaceae* contains over 188 species, most of which are saprophytic non-tuberculous mycobacteria (NTM). In wildlife, a variety of different NTM can be found, with different reports about their pathogenic potential. A pathogenic member of NTM is *Mycobacterium avium* ssp. *paratuberculosis* (MAP), which can infect farmed and wild ruminants. It causes paratuberculosis which is an economically important chronic disease. Infected farm animals are considered to be the source of infection in wild animals. Wildlife, on the other hand, is thought to be a reservoir for certain members of the *Mycobacterium tuberculosis* complex (MTBC), such as *M. caprae*, which causes tuberculosis in cattle and red deer.

**Methods:**

Switzerland implemented a surveillance program for tuberculosis in wild animals in 2014. Here, we describe the results from the mycobacterial culture of lymph node samples collected from red deer, roe deer, chamois, ibex, and badgers collected within this surveillance program from 2020 to 2022. Overall, samples from 548 animals were checked macroscopically for tuberculosis-like lesions.

**Results:**

In total, 88 animals (16.1%), which either had lesions in their lymph nodes or were male and aged older than 5 years, were investigated using mycobacterial culture. In total, 25 animals (28.4%) were positive for NTM, while no MTBC was detected. The most often identified NTM was *M. vaccae*, followed by *M. avium*. Most animals positive for NTM did not show any macroscopic lesions. Furthermore, MAP was isolated from the head lymph nodes of two male red deer. Neither of the two MAP-positive animals had any macroscopic lesions in their head lymph nodes or any other signs of disease.

**Discussion:**

The shooting sites of the two MAP-positive animals were located in Alpine pastures used for grazing of cattle during summer, which confirms that species transmission can occur when contaminated pastures are used by different species. In agreement with other studies, the occurrence of MAP in red deer was quite low. However, so far, MAP was mostly isolated from feces and intestinal lymph nodes of wild animals. This is the first detection of MAP in the head lymph nodes of red deer in Switzerland.

## 1 Introduction

The family *Mycobacteriaceae* contains over 188 species, most of which are saprophytes found in the environment. Saprophytic mycobacteria belong to the group of non-tuberculous mycobacteria (NTM), and many of them are opportunistic pathogens (1). In wild animals, a variety of different NTM can be found. Frequently found species include *Mycobacterium* (*M*.) *non-chromogenicum, M. peregrinum*, and *M. scrofulaceum* (2–4). In Switzerland, only data regarding wild boars are available, in which *M. avium* ssp. *hominissuis* is found most frequently, followed by *M. nonchromogenicum* (5). While some infected animals appear healthy without any visible lesions, others have granulomatous lesions in their lymph nodes (2, 3). However, there are also obligate pathogenic NTM, such as *Mycobacterium avium* ssp. *paratuberculosis* (MAP), which causes the chronic disease paratuberculosis in ruminants. In cattle, the disease causes diarrhea, weight loss, and reduced milk yield, which leads to economic losses (1). Furthermore, cattle are thought to be the source of infections of MAP in red deer (6). Infected red deer can develop typical clinical signs of paratuberculosis; however, they can also remain asymptomatic (7). The mean prevalence of MAP in wildlife in different countries is low at only 2.4%, however, there is quite a big range, i.e., from 0 to 100% (8). In Swiss red deer, the prevalence of MAP seems to be low as well (9).

Another important group of mycobacteria is the *Mycobacterium tuberculosis* complex (MTBC), which causes tuberculosis in humans and animals (1). Bovine tuberculosis is caused by *M. bovis* and *M. caprae*. However, other species such as *M. tuberculosis* and *M. microti* can also infect ruminants (10, 11). Due to its zoonotic potential and economic losses, many countries have introduced eradication programs for bovine tuberculosis (10). While Switzerland is officially free of bovine tuberculosis, the risk of recurrence of the disease remains as it was the case in two different outbreaks in 2013. One of these cases was caused by *M. bovis*, which seems to have persisted within a herd of animals for 15 years (12, 13). The other outbreak was caused by the *M. caprae* Lechtal genotype, which is endemic in Austrian red deer and dairy cattle (12). While *M. caprae* was not found in red deer in Switzerland before, there is a hot-spot area of red deer infected with *M. caprae* in Vorarlberg, Austria, close to the Swiss border (14, 15). As members of the MTBC can survive in the environment for a long period of time and indirect oral transmission is possible (16), it seems likely that the Swiss cattle got infected while summering on Austrian pastures contaminated with *M. caprae* (12, 14).

Red deer are considered reservoir hosts for tuberculosis and pose a source of infection for cattle (17). As a result of the outbreak of *M. caprae* in Swiss cattle in 2013, a surveillance program for tuberculosis in wild animals in Switzerland was implemented in 2014 by the Swiss Federal Food Safety and Veterinary Office and the Swiss cantons Grisons and St. Gallen and the Principality of Liechtenstein (12). As various wild animals, such as badgers, wild boar, red fox, and cervids, can be infected with bovine tuberculosis (18, 19), the program includes a risk-based approach, for which dead or diseased wildlife (red deer, chamois, ibex, roe deer, wild boar, foxes, and badger) are examined throughout the year. Furthermore, the program includes a random sampling approach, which examines red deer shot during hunting season. The sampling region covers the Swiss cantons of Grisons and St. Gallen and the Principality of Liechtenstein. Retropharyngeal and mesenteric lymph nodes are the primary sites of infection for tuberculosis, and lesions occur most often in these two lymph nodes (14). Hence, retropharyngeal and mandibular lymph nodes from all animals were collected and for the risk-based group, other lymph nodes, such as thoracic or mesenteric lymph nodes, were collected as well.

This study analyzes the data from the surveillance program from 2020 to 2022. While no mycobacteria from the MTBC were detected, various NTM, including MAP, were isolated. To the best of our knowledge, this is the first detection of MAP in the head lymph nodes of red deer in Switzerland. Furthermore, while NTM were detected in red deer in Switzerland before (15), this is the first study that identifies different NTM species found in red deer and wild animals other than wild boars in Switzerland.

## 2 Materials and methods

### 2.1 Sample collection

The samples used in this study were collected for a surveillance program for tuberculosis in wild animals in Switzerland, which was implemented in June 2014. In this study, only samples collected from 2020 to 2022 are included. This is due to the fact that starting from 2020, lymph nodes from male animals older than 5 years were directly analyzed using mycobacterial culture, irrespective of their macroscopic appearance. This change was implemented to increase the sensitivity of the surveillance, as older male animals are at a higher risk of infection with tuberculosis (20). In the majority of cases, lymph nodes were collected by veterinarians in the slaughterhouse, otherwise by hunters in the field.

Overall, for the risk-based approach, lymph node and organ samples were collected from 35 wild animals, as shown in Table 1. The random sampling group contained lymph node samples from 513 red deer, as shown in Table 2. Retropharyngeal and mandibular lymph nodes from all animals were collected. Additionally, for the risk-based group, organs with lesions and their associated lymph nodes, such as thoracic or mesenteric lymph nodes, were collected as well. However, in a few cases of the risk-based approach, no head lymph nodes were collected; thus, in these cases, only the organs with lesions and their associated lymph nodes were examined.

**Table 1:** Number of samples collected from different wild ruminants and badgers for the risk-based approach.

	**Red deer**	**Roe deer**	**Chamois**	**Ibex**	**Badger**	**Total**
Male < 5 years	3	2	1	0	0	6
Male > 5 years	10	0	1	1	0	12
Female < 5 years	3	0	0	0	0	3
Female > 5 years	7	0	0	0	0	7
Age and/or sex unknown	1	1	2	0	3	7
Total	24	3	4	1	3	35

**Table 2:** Number of samples collected from red deer for the random sampling approach.

	**Male**	**Female**	**Sex unknown**	**Total**
< 5 years	87	151	0	238
> 5 years	65	201	0	266
Age unknown	4	3	2	9
Total	156	355	2	513

### 2.2 Macroscopic evaluation of the lymph nodes

Upon arrival in the laboratory, the tissue surrounding the lymph nodes was removed. The lymph nodes were then stored at −20°C until further processing. For the macroscopic examination, the lymph nodes were thawed and cut into 1.0-mm thick slices and then examined for macroscopic lesions using a magnifying lens. Typical lesions of tuberculosis in red deer consist of purulent abscesses, though lesions can also be granulomatous (14, 21). If no lesions were found, the sample was considered negative. Lymph nodes from male animals aged older than 5 years were not macroscopically examined in detail, since they were directly processed for mycobacterial culture.

### 2.3 Mycobacterial culture

Culture of mycobacteria was conducted for all lymph nodes with suspicious lesions and for all male animals aged older than 5 years. For the mycobacterial culture for each animal, all the received lymph nodes were pooled. First, the lymph nodes were cut into small pieces and homogenized using a disperser (T18 digital ULTRA-TURRAX^®^, IKA, Staufen, Germany). Following, decontamination of the homogenized lymph nodes was conducted to get rid of organic contamination and normal flora. The decontamination was carried out according to a protocol described by the WHO in the Laboratory Services in Tuberculosis Control Part III (22). For that purpose, 4% H_2_SO_4_ was added. After 15 min of incubation, it was neutralized by the addition of 1N NaOH. The sample was then washed with PBS and centrifuged for 15 min at 3000 g. The sediment was resuspended in PBS buffer and streaked on a rigid growth medium for mycobacterial culture (Löwenstein–Jensen and Stonebrink media, Artelt–Enclit, Rötha OT Oelzschau, Germany) and additionally to the liquid mycobacteria growth indicator tube (MGIT™, Becton Dickinson, Allschwil, Switzerland), where BD MGIT™ growth supplement and BD MGIT™ PANTA™ antibiotic mixture were added. To support the growth of *M. bovis* and MAP, pyruvate (Carl Roth AG, Arlesheim, Switzerland) and mycobactin (Innovative Diagnostics, Grabels, France) were added to the MGIT™.

The mycobacterial cultures were incubated at 37°C for 7 weeks. The Löwenstein–Jensen medium and Stonebrink medium were checked for growth once a week. The MGIT™ was incubated in the BD BACTEC™ MGIT™ 320 system, which conducts hourly fluorescence measurements. If the BD BACTEC™ MGIT™ 320 system measured a positive result, Ziehl–Neelsen staining was performed to confirm the growth of acid-fast bacilli. Afterward, the samples were subcultured on a Middlebrook 7H10 solid growth medium (Becton Dickinson, Allschwil, Switzerland), supplemented with an in-house PANTA antibiotic mixture. After the growth of mycobacteria, real-time PCR (*artus M. tuberculosis* RG PCR Kit, Qiagen, Hilden, Germany) was performed to exclude the presence of MTBC. MALDI-TOF MS (Bruker Daltonics, Billerica, MA, USA) was used for the identification of mycobacteria. In two cases, MAP was detected, which was confirmed with PCR (ID Gene™ Paratuberculosis Duplex, Innovative Diagnostics, Grabels, France). The two MAP strains were further characterized by MIRU-VNTR, PCR, and restriction enzyme digestion for the identification of C-type or S-type strain, as previously described (23).

### 2.4 Histology

Different tissues from six animals with macroscopic lesions were fixed in 4% formalin (Table 3). The following day, formalin-fixed tissues were trimmed and embedded in paraffin, and histological tissue sections of 2–3 μm thickness were cut and stained with hematoxylin and eosin (HE). Slides were then assessed under a light microscope.

**Table 3:** Animals with macroscopic lesions in their lymph nodes or other organs and results from histology and mycobacterial culture.

**Sampling approach**	**Animal**	**Macroscopic lesions**	**Histology**	**Result of the mycobacterial culture**
Random sampling	Red deer, f > 5 years	Pus-filled lymph node	-	Negative
	Red deer, f > 5 years	Pus-filled palatine tonsil	Moderate multifocal suppurative necrotizing tonsillitis	Negative
	Red deer, f > 5 years	Pus-filled lymph node	Slight eosinophilic lymphadenitis	Positive for *M. nonchromogenicum*
Risk-based sampling	Red deer, sex and age unknown	Nodules under the skin	-	Negative
	Red deer, f < 5 years	Pus-filled kidney	Renal carcinoma	Negative
	Roe deer, sex and age unknown	Lung abscess	-	Negative
	Chamois, sex and age unknown	Abscess on the head, abscessing pneumonia	Multifocal to diffuse granulomatous and eosinophilic pneumonia with parasite cut sections	Negative
	Chamois, sex and age unknown	Inconclusive macroscopic evaluation of the lymph nodes, lesions in the lungs	-	Negative
	Chamois, m > 5 years	Suspicious lesions in lymph nodes, lesions in the lungs	Multifocal granulomatous and eosinophilic pneumonia with parasite cut sections	Positive for *M. vaccae*
	Badger, sex and age unknown	Calcifications in the prescapular lymph nodes, lesions in the spleen, liver, kidney, and lungs, positive for canine distemper virus	Multifocal granulomatous and eosinophilic pneumonia with parasite cut sections	Positive for *M. avium*

## 3 Results

### 3.1 Macroscopic evaluation of the lymph nodes

In the risk-based approach, seven animals (two red deer, one roe deer, three chamois, and one badger) had macroscopic lesions in their lymph nodes or other organs, as shown in Table 3. One of these animals (chamois) was male and aged older than 5 years. In the random sampling group, three female red deer had purulent lesions in their lymph nodes. None of the other animals had any macroscopic lesions.

### 3.2 Mycobacterial culture

In the risk-based approach, mycobacterial culture was carried out in 20 out of 35 samples (57.1%). In total, 12 of these animals were male animals aged 5 years or older (Table 1; 10 red deer, 1 chamois, and 1 ibex). Additionally, the mycobacterial culture of lymph nodes with macroscopic lesions of one roe deer, two chamois, one badger, and two red deer was performed (Table 3). Furthermore, the lymph nodes of two badgers without macroscopic lesions were included in the culture, as badgers do not always show typical lesions in their lymph nodes (24, 25). Positive cultures for NTM were detected in six samples (6 of 20, 30%; Table 4). Only two animals with macroscopic lesions had a positive result in the mycobacterial culture (Table 3). One of them, a male chamois aged older than 5 years with macroscopic lesions in the lymph nodes and pneumonia with parasites, was positive for *M. vaccae*. The other culture-positive animal with lesions was a badger, which had lesions in the lymph nodes and various organs and was also found to be infected with the canine distemper virus and lungworms. This badger was positive for *M. avium*. The other two badgers, which did not have any lesions, were also found to be positive for *M. avium* (Table 4). There were two more culture-positive animals, namely, two male red deer aged older than 5 years without any macroscopic lesions. These two animals were infected with *M. diernhoferi* and a mixed infection with two different NTM, respectively.

**Table 4:** Samples submitted to mycobacterial culture with the identified mycobacterial species.

**Sampling approach**	**Animal species**	**Number of animals tested with mycobacterial culture**	**Number of mycobacterial culture positive samples**	**Mycobacteria identified**	**Number of strains identified**
Random sampling	Red deer	68	19	*M. avium* ssp. *paratuberculosis*	2
				*M. avium* ssp. *hominissuis*	2
				*M. diernhoferi*	1
				*M. nonchromogenicum*	2
				*M. porcinum*	1
				*M. terrae* complex	1
				*M. vaccae*	7
				NTM	3
Risk-based sampling	Red deer	12	2	*M. diernhoferi*	1
				NTM (mixed-infection)	1
	Roe deer	1	0	-	-
	Chamois	3	1	*M. vaccae*	1
	Ibex	1	0	-	-
	Badger	3	3	*M. avium*	2
				*M. avium* ssp. *hominissuis*	1
	Total	20	6		
Total		88	25		

In the random sampling group, 68 out of 513 samples (13.3%) were tested with mycobacterial culture. In total, 65 of the samples belonged to male animals older than 5 years, without any obvious macroscopic lesions in their lymph nodes. Overall, 18 of these male animals had a positive culture. Only one of the female animals with macroscopic lesions in its lymph nodes had a positive culture for NTM (*M. nonchromogenicum*). Altogether, a positive culture for NTM was detected in 19 samples (19 of 68, 27.9%; Table 4).

Overall, 548 samples were analyzed, of which 88 samples (16.1%) were tested with the mycobacterial culture and 25 samples were positive (25 of 88, 28.4%). The different species identified are listed in Table 4. No animal had a positive culture for the *Mycobacterium tuberculosis* complex. Overall, *M. vaccae* was identified most often (8 of 25, 32%). The second most identified NTM was *M. avium* (7/25, 28%), which was further identified as *M. avium* ssp. *hominissuis* in three cases and MAP in two cases. Both MAP-positive animals were male red deer from the random sampling group, aged 8 and 13 years, respectively. The samples from these two animals were taken in the slaughterhouse. Neither of these two animals had any macroscopic lesions in their head lymph nodes or any other signs of disease. However, the shooting sites of the two animals were located in Grisons near Alpine pastures, which were used for summering of cattle. Both MAP strains were identified as C-type belonging to the INMV1 profile. Furthermore, in four culture-positive cases, the species could not be identified due to poor growth of the mycobacteria. These strains were classified as NTM. Additionally, in one of these cases, there was a mixed infection with two different NTM.

### 3.3 Histology

In all tissue samples histologically examined (Table 3), no lesions interpreted as induced by NTMs could be discerned. In the risk-based approach, four animals were histologically examined. Three animals, whose lung tissue was assessed, had pneumonia induced by lungworms. Two of these animals were also positive in the mycobacterial culture, namely, for *M. vaccae* and *M. avium*, respectively. One animal with a renal carcinoma interpreted as an incidental finding had no histological lesions in the lymph node and was negative in the mycobacterial culture. In the group from the random sampling, histology was carried out in two animals. One animal had a suppurative necrotizing tonsillitis, with a negative mycobacterial culture. The other animal had a slight eosinophilic lymphadenitis, which was most likely induced by parasitic infestation. This animal was also positive for *M. nonchromogenicum*.

## 4 Discussion

Our study is the first to describe the isolation of MAP from the head lymph nodes of red deer in Switzerland. Furthermore, the isolation of various NTM provides an overview of different NTM species found in Swiss wild ruminants and badgers. In concordance with other studies, no MTBC was detected.

MAP was isolated from the head lymph nodes of two red deer. To the best of our knowledge, this is the first isolation of MAP from red deer in Switzerland. However, there is a reported case of MAP in a roe deer from Switzerland, which had severely enlarged lymph nodes and a case history of severe diarrhea (26). Another study from Switzerland that tested fecal samples from different wild ruminants was not able to cultivate MAP, suggesting only a low occurrence of MAP in Swiss wild ruminants (9). Recently, we detected another MAP-positive red deer in Switzerland (data not shown), which supports the low occurrence of MAP and also suggests that the other two MAP-positive red deer were not just a coincidental finding. Generally, the MAP prevalence in wildlife in other countries is also quite low, though the prevalence can be rather high in some countries, for example in Northern Italy and Spain (8, 27–29). However, the diagnosis of paratuberculosis is complicated by its long incubation period, the intermittent shedding of MAP in feces, and the lack of completely reliable diagnostic tests (30, 31), which might lead to an underestimation of the MAP prevalence. It is suggested that cattle are the source of infection for wild animals, although transmission from wild animals to cattle is possible as well (6). Interestingly, the two MAP-positive red deer in our study were both shot close to Alpine pastures used for grazing of cattle during summer. Thus, it is possible that the pastures are contaminated with MAP and pose a source of infection for red deer. The two MAP strains in our study were identified as C-type strains; the pattern found by MIRU-VNTR was INMV1. INMV1 is the predominantly found profile in Swiss cattle (23). Neither of the two MAP-positive animals had any clinical signs of disease or any macroscopic lesions. Other studies also found that the majority of MAP-infected red deer are asymptomatic and without macroscopic lesions (7, 28). Hence, it is possible that we missed some MAP-positive animals in our study since we did not conduct mycobacterial culture from all sampled animals. Furthermore, young red deer aged 8–15 months seem to be more susceptible to MAP infection than older animals and are thus more likely to develop clinical signs (7, 32). This might also explain the lack of clinical signs in the two MAP-positive red deer from our study, since the animals were already 8 and 13 years old, respectively. While vertical transmission of MAP is possible, the typical route of infection is fecal–oral. After ingestion, the pathogen is found within macrophages in the submucosa of the ileocecal area and its surrounding lymph nodes (1). Hence, typical samples for the diagnosis of MAP include feces, intestinal lymph nodes, the ileocecal valve, or intestines (8). However, in our study, we detected MAP in the head lymph nodes. A likely explanation for this would be that the head was the primary entry site of MAP (33), as head lymph nodes are also described as the primary site of infection for other mycobacteria (14).

Apart from MAP, we detected various NTM in different wild ruminants and badgers. The most often identified species was *M. vaccae*, which we detected in red deer and chamois. In Switzerland, *M. vaccae* was detected in wild boar before (5). In other countries, *M. vaccae* was also isolated from red deer (2, 3, 34). For *M. vaccae*, there is no histological evidence for pathogenicity in deer (34). In our study, most animals infected with this pathogen did not show any lesions in their lymph nodes, which supports the non-pathogenic nature of this agent. However, a chamois infected with *M. vaccae* did have suspicious lesions in its lymph nodes. While we cannot rule out other pathogens as the cause of these lesions, it is possible that *M. vaccae* can cause a disease under certain conditions. Similar results were observed for *M. nonchromogenicum*, for which only one animal showed macroscopic lesions, while, according to de Lisle, there is also no histological evidence for pathogenicity in deer (34). The second most identified NTM in our study was *M. avium*, which could be further specified as *M. avium* ssp. *hominissuis* in three cases and was found in red deer and badgers. In Swiss wild boars, *M. avium* ssp. *hominissuis* was the most often identified NTM (5); thus, it seems to be widespread among wildlife in Switzerland. Furthermore, *M. avium* ssp. *hominissuis* was also frequently detected in Slovenia (3). In our study, only one of the badgers infected with *M. avium* had lesions in the lymph nodes and other organs. However, this badger also tested positive for the canine distemper virus, which might have weakened the immune system, thus making the badger more susceptible to the mycobacterial infection. According to other studies, *M. avium* can be found in animals with and without lesions (3, 5, 35). In our study, animals infected with all other NTM species did not show any macroscopic lesions. Hence, it seems that most NTM are not pathogenic for wildlife, although some NTM might cause disease under certain conditions. This is similar to the findings in humans, where many NTM are considered opportunistic pathogens that mainly cause diseases in immunocompromised people (36). However, more studies are needed to determine the pathogenic potential of different NTM in animals. The prevalence and variety of different NTM species detected in Swiss wild boars were much higher than in our study, and apart from *M. porcinum* and MAP, all NTM species isolated in our study were detected in Swiss wild boars before (5). Thus, it seems likely that the prevalence and variety of NTM species found in wildlife are higher than we detected, and that the same NTM species can be found in different wild animals. Other countries also reported that a high variety of NTM was found in different wild animals, and apart from *M. porcinum*, all NTM species isolated in our study were detected in other countries before (2, 3, 34, 35). Furthermore, there are most likely still unknown NTM species, which could explain, why we could not identify all NTM in our study. This variety of NTM found in animals can be explained by the occurrence of NTM in the environment. Through contact with different sources of NTM, such as soil, dust, or water, animals can get infected (36).

As a conclusion, various NTM can be found in different wild animals in Switzerland. It seems that most of these NTM are not obligate pathogens, though some might act as opportunistic pathogens.

## Data availability statement

The original contributions presented in the study are included in the article/supplementary material, further inquiries can be directed to the corresponding author.

## Ethics statement

Ethical approval was not required for the study involving animals in accordance with the local legislation and institutional requirements because the animals investigated in this study were either shot during regular hunting or found dead from a natural cause.

## Author contributions

JL: Formal analysis, Visualization, Writing—original draft. UF: Data curation, Investigation, Methodology, Writing—original draft. CP: Conceptualization, Resources, Writing—review & editing. MH: Data curation, Formal analysis, Investigation, Writing—original draft. SaS: Conceptualization, Methodology, Project administration, Resources, Supervision, Writing—review & editing. SiS: Data curation, Formal analysis, Investigation, Writing—review & editing.
